# Immunocytochemistry Improving the Diagnosis of* Trichomonas vaginalis* Infections

**DOI:** 10.1155/2017/5642535

**Published:** 2017-03-23

**Authors:** Thaisa Helena S. Fonseca, Fabrício M. Silva Oliveira, Marina Alacoque, Marcella Israel Rocha, Henrique Vitor Leite, Joseph F. Guimarães Santos, Haendel G. N. O. Busatti, Marcelo Vidigal Caliari, Maria Aparecida Gomes

**Affiliations:** ^1^Department of Parasitology, Institute of Biological Sciences, Universidade Federal de Minas Gerais, Belo Horizonte, MG, Brazil; ^2^Department of Pathology, Institute of Biological Sciences, Universidade Federal de Minas Gerais, Belo Horizonte, MG, Brazil; ^3^Department of Gynecology and Obstetrics, Universidade Federal de Minas Gerais, Belo Horizonte, MG, Brazil; ^4^IPSEMG Hospital, Belo Horizonte, MG, Brazil; ^5^Universidade de Itaúna, Itaúna, MG, Brazil

## Abstract

The aim of this study was to evaluate the immunocytochemistry (ICC) to diagnose trichomoniasis, particularly asymptomatic infections. By culture serial dilutions, ICC was able to detect 1 trophozoite/mL, while the culture was positive up to 100 trophozoites/mL. The ICC in vivo detection capability was assessed in vaginal secretions of mice experimentally infected and in vaginal swabs from asymptomatic HIV-positive pregnant women compared with culture. All vaginal secretion samples from mice were positive according to both methods. Swabs from fifty-five asymptomatic women were positive in four (7.27%) of them by culture. Beyond these four, another ten (25.45%) women were positive by immunocytochemistry, proving their higher sensitivity (*p* = 0.002), noticing 3.5 times more positives. ICC had better performance in both successive dilutions as in asymptomatic women, showing higher sensitivity and specificity. In this way, its facility of execution and cost-effectiveness support its practicality, as a routine procedure to diagnose trichomoniasis not only when the parasite load is lower but probably in all clinical scenarios.

## 1. Introduction

Trichomoniasis accounts for 280 million of new cases of sexually transmitted infections worldwide [1]. It is recognized by its morbidity and as a facilitator of human immunodeficiency virus (HIV) transmission and acquisition, since 24% of HIV infections are directly correlated with trichomoniasis [2, 3].

Trichomoniasis presents a great variety of clinical manifestations, with the signs and symptoms depending upon the individual condition of each patient, the pathogenicity of the parasite, and the number of infecting trophozoites [4]. In men, the infection is mostly asymptomatic and they are subsequently regarded as the greatest disseminator of the disease [5]. Symptomatic cases are observed in females, with the occurrence of itching, vaginal discharge, vulvitis, erosion, edema, and hemorrhagic areas in the cervical wall. Important complications associated with the disease during pregnancy are often observed [6, 7].

Asymptomatic patients represent 50% to 70% of infected individuals resulting in a higher potential of parasite transmission [8]. Therefore, it is important to highlight the power of trichomoniasis in the amplification of HIV transmission [9, 10]. In this context, a laboratory investigation, mainly in asymptomatic infections, is essential for trichomoniasis control. In the laboratory routine, tests use techniques which have relatively low sensitivity, such as the direct examination of wet and stained preparations [11], culminating with the underestimated prevalence of the disease. Other FDA approved techniques are available for trichomoniasis diagnosis; among these nucleic acid amplification tests (NAATs) have shown high sensitivity and specificity [12]. However, the expensive cost of this diagnostic method makes it unaffordable for developing countries like Brazil [13]. Culture has a greater sensitivity (75 to 96%), being the main test used to confirm suspected cases, especially in developing countries [14]. However, the material for examination must be collected and cultivated properly and a few days are required for the identification of the parasite, which can compromise the disease control [11].

The aim of this study was to evaluate the viability of immunocytochemistry (ICC), as an alternative tool not only to diagnose trichomoniasis but especially to diagnose lower parasitic loads, such as in asymptomatic infections.

## 2. Materials and Methods

### 2.1. Study Design

This study was composed of two phases. The first is an experimental test in Balb/c mice, to determine the ICC's ability to identify true positives and negatives. The second is a cross-sectional study in asymptomatic HIV-positive women comparing ICC with culture to diagnose* Trichomonas vaginalis*.

In literature, we found prevalence ranging from 3 up to 53%. The average prevalence was 25%. Therefore, we adopted the value of the standard deviation of this prevalence for the accuracy of the sample calculation, whose value was 12% [15–17]. Thus, a sample size of 50 individuals was estimated. In all study phases, the culture was used as a pattern.

### 2.2. Cultivation and Growth Conditions

The* T. vaginalis *VPFS strain from Caratinga, MG, Brazil, was isolated in 2013 from a 26-year-old female symptomatic patient. Trophozoites of this strain were cultivated in a sterile glass tubes (Pyrex®) containing YI-S culture medium and kept in a bacterial growth chamber at 37°C. Subcultures performed every 48−72 h ensured maintenance of the parasite in the exponential growth phase. For cultivation of samples from vaginal swabs, antibiotics and antifungal were added [18].

### 2.3. Dilution Test

First, we estimated in vitro ICC capability to detect* T. vaginalis *trophozoites, compared with culture, using a series of 10-fold dilutions from 1000 trophozoites/mL.

### 2.4. Sampling of Vaginal Secretions of Mice

Afterwards, the ICC in vivo capability to detect* T. vaginalis* was assessed in vaginal secretions of mice experimentally infected compared with the culture. A combination of pretreatment in mice with estradiol and dexamethasone, with or without antibiotics, resulted in a significantly higher infection rate (60−100%) by* T. vaginalis* [19]. Thus, ten 7-week-old female Balb/c mice, weighing approximately 15 g, received 50 *μ*g of estradiol valerate (Delestrogen, JHP Pharmaceuticals, Rochester, MI) in suspension of 100 *μ*L of sesame oil (Sigma-Aldrich, St. Louis, MO) subcutaneously 9 and 2 days before infection. These animals also received 10 mg/kg of disodium dexamethasone phosphate (APP Pharmaceuticals, Schaumburg) diluted in 100 *μ*L of PBS intraperitoneally daily for 4 days prior to* T. vaginalis* infection and up to 6 days after infection.

A combination of antibiotics was used to reduce commensal vaginal microbiota. Therefore, each animal received intraperitoneally vancomycin hydrochloride (Sigma-Aldrich) and streptomycin sulfate (Sigma-Aldrich) suspended in 100 *μ*L of PBS for 4 days before challenge and up to 6 days after challenge. Animals also received trimethoprim orally at 0.4 mg/mL in ad libitum water for the same period. The animals were intravaginally infected with 1 × 10^7^ trophozoites. The use of animals was approved by Animal-Care Ethics Committee of the UFMG (COEP Protocol # 364.230).

### 2.5. Sampling of Vaginal Secretions of Women

Considering the strong association between trichomoniasis and HIV [2, 9], we enrolled in this study HIV-positive pregnant women with no signs of trichomoniasis on gynecologic examination. These women were treated for HIV at the Jenny de Andrade Faria Institute in the Clinical Hospital-UFMG with research design, protocol, and informed consent approved by the UFMG Ethics and Research Committee (Protocol # 413/2015). The women who volunteered to participate in this research freely signed the informed consent having their identities preserved. The vaginal samples were collected using sterile swabs. Part of the material collected was cultivated in glass tubes containing YI-S medium with addition of antibiotics and antifungals, as previously described [19]. Another portion of the material was used to make the smears to be used by the ICC technique.

### 2.6. Production of Anti-*T. vaginalis* Antibody

The production of the polyclonal anti-*T. vaginalis *antibody followed a protocol previously described [20] with some modifications. Briefly, 1 × 10^7^ trophozoites of* T. vaginalis *(VPFS strain) were concentrated by centrifugation. The pellet was resuspended in 1 mL PBS (pH 7.2) and sonicated (40 Hz frequency) for 1 min with three repetitions. After that, two Wistar, 10-week-old female rats weighing approximately 270 g were subcutaneously inoculated with 0.5 mL of sonicate emulsified in Freund's complete adjuvant (Thermo Fisher Scientific, USA). Fifteen days after the first inoculation, the animals received a booster with the material obtained as described for the first immunization without the adjuvant. Ten days after, these animals were exsanguinated through the ribcage for blood collection. Subsequently, the blood was centrifuged in order to obtain the anti-*T. vaginalis *antibody and kept at −20°C until the immunocytochemical reaction was carried out.

### 2.7. Immunocytochemical Reaction

Smears from culture serial dilutions or vaginal secretions from mice and women followed the same protocol described previously with some modifications [21]. The smears were fixed in alcohol 90% and washed in phosphate buffered saline (PBS; pH 7.2) for 5 min, and endogenous peroxidase activity was eliminated by incubating the slides in 0.2% hydrogen peroxide solution (H_2_O_2_) for 20 min. The buffer used for the dilution of the antibodies was bovine serum albumin (2%). Unspecific binding was blocked by goat serum diluted 1 : 40 for 40 min at room temperature (26°C). Smears were incubated for 40 min with polyclonal anti-*T. vaginalis *antibody diluted at 1 : 300, followed by biotinylated goat anti-rat IgG and streptavidin, both diluted 1 : 100 and purchased from Zymed Laboratories, San Francisco, CA, USA. The color was revealed using a 0.05% diaminobenzidine solution and 0.2% H_2_O_2_ and the slides were counterstained with diluted Harris's haematoxylin. Smears of* T. vaginalis* trophozoites originated from cultures were used as a positive control. As negative control, swabs from noninfected animals were used. For ICC negative control, the primary antibody was substituted by PBS in the reactions with swabs from infected animals and women.

### 2.8. Data Analysis

Data were expressed as percentages and for paired comparisons, McNemar's test was used. To measure the concordance between culture and ICC we used kappa test. To determine the diagnostic value of ICC we calculated the sensitivity, sensibility, and predictive values, considering ICC as diagnosis pattern. Statistical analyses were performed using the SPSS 23.0 software package (SPSS Inc., Chicago, IL, USA) and a 95% confidence interval was used.

## 3. Results

### 3.1. Performance of Culture and Immunocytochemistry In Vitro and In Vivo Assays

The ICC was able to detect 1 trophozoite/mL, while culture was positive up to 100 trophozoites/mL in serial dilutions.

**Figure 1:**
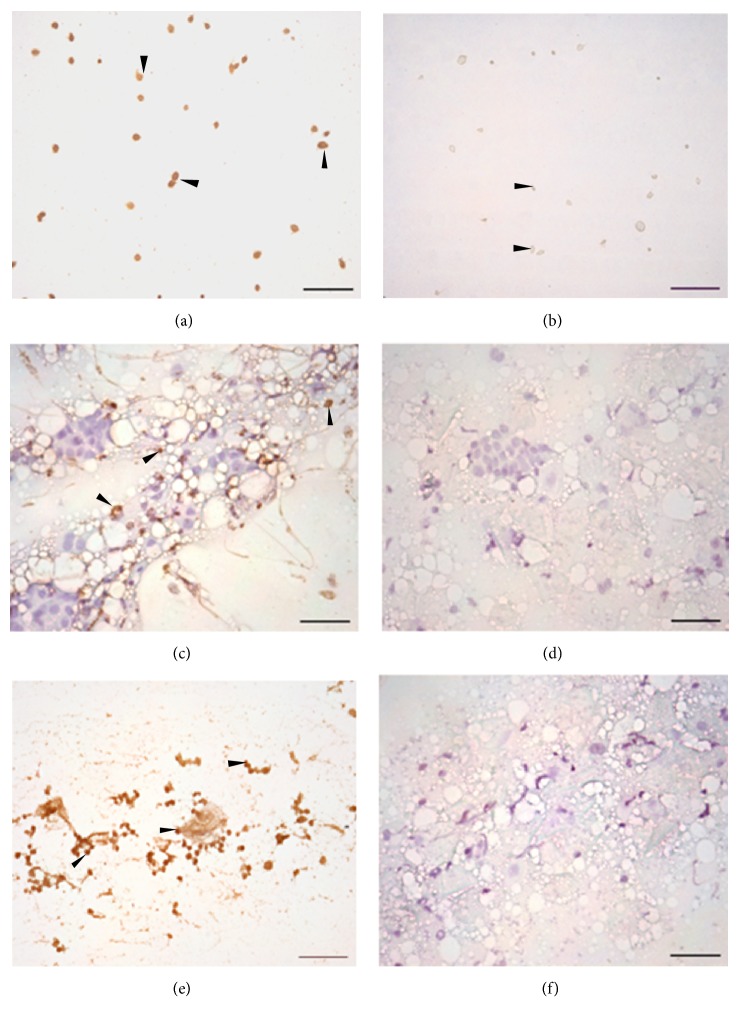
Immunocytochemical reaction for* Trichomonas vaginalis *trophozoites. (a)* T. vaginalis *culture as positive control. (b)* T. vaginalis *culture with primary antiserum substituted by PBS for negative control. (c) Vaginal smears from mice in the fourth day of infection, showing specific marking for* T. vaginalis *trophozoites (arrowheads). (d) Vaginal smears from mice used as negative control. (e) Vaginal smears of a HIV-positive woman showing positive reaction for* T. vaginalis *trophozoites (arrowheads) (f) Vaginal smears of HIV-positive woman used as negative control. Bar: 100 *μ*m.

The smears from cultures, used as positive control in ICC, revealed a dark brown color resulting from the binding of the polyclonal anti-*T. vaginalis *antibody and precipitation of the diaminobenzidine, enabling visualization of the parasite in its characteristic piriform shape along with its prominent flagella adhered to epithelial cells (Figure 1(a)). In contrast, the slides, used as negative control, showed only a violet color conferred by the Harris hematoxylin (Figure 1(b)).

Vaginal secretions of mice were evaluated until the 8th day of infection. All infected animals were positive by cultivation and ICC in the 2nd day of infection. On the 4th day of infection, ICC detected* T. vaginalis* in 100% of the infected animals, whereas the culture was negative in all of them (Table 1). On the 8th day of infection, the culture remained negative but the ICC determined a low parasitic load.

In ICC, trophozoites of* T. vaginalis* showed specific markings, as described above for positive controls (Figure 1(c)). No marking was observed on the vaginal smear of infected mouse used as negative control (Figure 1(d)). Two cultures became positive after the fifth day of incubation.

### 3.2. Performance of Culture and Immunocytochemistry in HIV-Positive Women

Fifty-five women that agreed to sign the informed consent were enrolled in this study. The vaginal secretions were positive in four cultures (7.27%). The ICC identified the parasite in these same four samples and in another ten (25.45%). ICC was significantly more sensitive (*p* = 0.002) to identify* T. vaginalis *in asymptomatic women than culture, noticing 3.5 times more positives (Table 2). In ICC, trophozoites were found randomly and well distributed in the mucus; many of them were adhered to vaginal epithelial cells (Figure 1(e)). Negative vaginal secretion to* T. vaginalis *showed only a violet color conferred by the Harris hematoxylin (Figure 1(f)).

As described above, in vitro and in vivo assays demonstrated that ICC had shown significantly greater sensitivity than culture in the detection of* T. vaginalis*. In this way, we compared the performance of the culture, considering ICC as gold standard test. Culture had a sensitivity of only 28.6%, despite a specificity of 100%, and positive predictive value of 28.6% and a negative predictive value of 100%. Thus, culture was unable to diagnose 10 (71.4%) out of 14 positive samples in ICC, where the parasite was evident.

In assessing the concordance between both tests, we found out a degree of agreement of 81.8%, with kappa of 0.374 (*p* < 0.001) (Tables 2 and 3). This shows that, despite significant agreement between the two tests, it is weak. This is due to the significant difference in sensitivity between the two tests, demonstrated by the higher rate of diagnosis made with immunocytochemistry, in relation to culture (7.3 × 25.5%).

## 4. Discussion

Recent studies showed that trichomoniasis increases the risk of HIV infection and may cause important complications during pregnancy. This situation has intensified interest in this parasite and highlighted the need for more sensitive diagnostic tests [6, 7].

Several tests are available in laboratory diagnosis for trichomoniasis, from basic microscopy to more complex tests, like PCR. The tests differ in their specificity and sensitivity, the complexity of their performance, and costs. Most of them require specific equipment and highly trained personnel, resulting in higher costs [11, 13]. Moreover, the low sensitivity to identify asymptomatic infections undermines many of them [9]. The culture, despite the lower sensitivity compared to NAAT, is still the technique of choice to evaluate suspected cases of trichomoniasis when wet mount is negative, mainly in developing countries [12]. Considering it, we choose culture to assess ICC as an alternative method for diagnosing trichomoniasis.

For ICC standardization and obtaining the true positives and negatives, we used Balb/c female mice intravaginally infected with* T. vaginalis *trophozoites.

Positive results were observed for both ICC and culture. However, cultures were positive only until the 2nd day of infection, remaining negative even following incubation as long as 7 days. The low parasite load observed in rodents may have contributed to the negative results observed in culture, which requires at least 100 trophozoites, to be positive [22]. These data are confirmed by the best performance of ICC to identify* T. vaginalis *in serial dilutions, which have no microorganisms or substances that could explain the absence of parasite growth in culture medium, confirming that the minimum concentration of the parasite is required to have positive results [11, 22].

For the application of ICC as an alternative diagnostic method, vaginal secretions were collected from HIV-positive pregnant women suspected to be infected by* T. vaginalis*. Immunocytochemistry was also significantly more sensitive to identify* T. vaginalis *in asymptomatic women than culture, noticing 70% more positives (*p* < 0.002), revealing its greater diagnostic power. These results are supported by the higher sensitivity of the technique to identify trophozoites in urine samples [21].

Techniques more sensitive than culture, such as identification of nucleic acids, are suggested as diagnosis of trichomoniasis. Among these techniques, there are NAAT, BD Probe Tec TV Qx Amplified, DNA Assay, OSOM Trichomonas Rapid Test, and Affirm VP III [12]. However, besides the cost, these techniques require specific equipment and trained personnel for its implementation, keeping them away from the reality of developing countries [13]. Thus, the ICC, being a known technique by most laboratories and requiring no special equipment for its execution, could be a strong candidate to replace the culture in the diagnosis of trichomoniasis.

## 5. Conclusion

The ICC high sensitivity and specificity to identify* T. vaginalis *infections, associated with its feasibility of execution and low cost, support its practicality as a routine procedure to diagnose trichomoniasis representing an important advance in the clinical and epidemiological approach to disease.

## Figures and Tables

**Table 1:** Diagnostic comparison of culture and immunocytochemistry for detection of *Trichomonas vaginalis *in animal model^*∗*^.

Mice vaginal sample	Culture	Immunocytochemistry	*p* value
Noninfected	0 (0.0)	0 (0.0)	—
Second day of infection	10 (100.0)	10 (100.0)	—
Fourth day of infection	0 (0.0)	10 (100.0)	<0.001

^**∗**^Ten 7-week-old female Balb/c mice were used for each group.

**Table 2:** Performance of immunocytochemistry for *Trichomonas* diagnosis in HIV-positive women asymptomatic (*n* = 55).

	Culture^**∗**^	Immunocytochemistry	*p* value^†^
Positive	4 (7.27)	14 (25.45)	0.002
Negative	51 (92.73)	41 (74.55)	—
Sensitivity (%)	—	100.0	—
Specificity (%)	—	80.4	—
Positive predictive value (%)	—	28.6	—
Negative predictive value (%)	—	100.0	—

^*∗*^Gold standard.

^†^McNemar test.

**Table 3:** Concordance between immunocytochemistry and culture.

	(*n*)	(%)	Concordance proportion (%)	Kappa	Concordance^*∗*^
Culture	4	7.02			
Immunocytochemistry	14	25.49	81.8	0.374	Low

^*∗*^ *p* < 0.001.

## References

[1] World Health Organization (2017). Global incidence and prevalence of selected curable sexually transmitted infections: 2008. *Reproductive Health Matters*.

[2] Mundodi V., Kucknoor A. S., Klumpp D. J., Chang T.-H., Alderete J. F. (2004). Silencing the ap65 gene reduces adherence to vaginal epithelial cells by *Trichomonas vaginalis*. *Molecular Microbiology*.

[3] Kissinger P. (2015). *Trichomonas vaginalis*: a review of epidemiologic, clinical and treatment issues. *BMC Infectious Diseases*.

[4] Petrin D., Delgaty K., Bhatt R., Garber G. (1998). Clinical and microbiological aspects of *Trichomonas vaginalis*. *Clinical Microbiology Reviews*.

[5] Harp D. F., Chowdhury I. (2011). Trichomoniasis: evaluation to execution. *European Journal of Obstetrics & Gynecology and Reproductive Biology*.

[6] Sood S., Kapil A. (2008). An update on *Trichomonas vaginalis*. *Indian Journal of Sexually Transmitted Diseases and AIDS*.

[7] Cudmore S. L., Garber G. E. (2010). Prevention or treatment: the benefits of *Trichomonas vaginalis* vaccine. *Journal of Infection and Public Health*.

[8] Wilkinson D., Abdool Karim S. S., Harrison A. (1999). Unrecognized sexually transmitted infections in rural South African women: a hidden epidemic. *Bulletin of the World Health Organization*.

[9] Shafir S. C., Sorvillo F. J., Smith L. (2009). Current issues and considerations regarding Trichomoniasis and human immunodeficiency virus in African-Americans. *Clinical Microbiology Reviews*.

[10] Hollman D., Coupey S. M., Fox A. S., Herold B. C. (2010). Screening for *Trichomonas vaginalis* in high-risk adolescent females with a new transcription-mediated nucleic acid amplification test (NAAT): associations with ethnicity, symptoms, and prior and current STIs. *Journal of Pediatric and Adolescent Gynecology*.

[11] Šoba B., Skvarč M., Matičič M. (2015). Trichomoniasis: a brief review of diagnostic methods and our experience with real-time PCR for detecting infection. *Acta Dermatovenerologica Alpina Pannonica et Adriatica*.

[12] Workowski K. A., Bolan G. A. (2015). Sexually transmitted diseases treatment guidelines. *MMWR Recommendations and Reports*.

[13] Gaydos C., Hardick J. (2014). Point of care diagnostics for sexually transmitted infections: perspectives and advances. *Expert Review of Anti-Infective Therapy*.

[14] Nye M. B., Schwebke J. R., Body B. A. (2009). Comparison of APTIMA *Trichomonas vaginalis* transcription-mediated amplification to wet mount microscopy, culture, and polymerase chain reaction for diagnosis of trichomoniasis in men and women. *American Journal of Obstetrics and Gynecology*.

[15] Spence M. R., Hollander D. H., Smith J., Mccaig L., Sewell D., Brockman M. (1980). The clinical and laboratory diagnosis of *Trichomonas vaginalis* infection. *Sexually Transmitted Diseases*.

[16] Bunnell R. E., Dahlberg L., Rolfs R. (1999). High prevalence and incidence of sexually transmitted diseases in urban adolescent females despite moderate risk behaviors. *Journal of Infectious Diseases*.

[17] Wendel K. A., Erbelding E. J., Gaydos C. A., Rompalo A. M. (2002). *Trichomonas vaginalis* polymerase chain reaction compared with standard diagnostic and therapeutic protocols for detection and treatment of vaginal trichornoniasis. *Clinical Infectious Diseases*.

[18] Diamond L. S., Clark C. G., Cunnick C. C. (1995). YI-S, a casein-free medium for axenic cultivation of *Entamoeba histolytica*, related *Entamoeba*, *Giardia intestinalis* and *Trichomonas vaginalis*. *Journal of Eukaryotic Microbiology*.

[19] Cobo E. R., Eckmann L., Corbeil L. B. (2011). Murine models of vaginal trichomonad infections. *American Journal of Tropical Medicine and Hygiene*.

[20] Costa C. A., De Brito K. N., Gomes M. A., Caliari M. V. (2010). Histopathological and immunohistochemical study of the hepatic lesions experimentally induced by *Entamoeba dispar*. *European Journal of Histochemistry*.

[21] O'Hara C. M., Gardner W. A., Bennett B. D. (1980). Immunoperoxidase staining of *Trichomonas vaginalis* in cytologic material. *Acta Cytologica*.

[22] Radonjic I. V., Dzamic A. M., Mitrovic S. M., Arsenijevic V. S. A., Popadic D. M., Zec I. F. K. (2006). Diagnosis of *Trichomonas vaginalis* infection: the sensitivities and specificities of microscopy, culture and PCR assay. *European Journal of Obstetrics & Gynecology and Reproductive Biology*.

